# RELIABILITY OF A NEW BAROPODOMETRY PLATFORM DURING BAREFOOT WALKING IN HEALTHY ADULTS

**DOI:** 10.1590/1413-785220253305e292018

**Published:** 2025-09-22

**Authors:** Silvia Iovine Kobata, Cynthia Rossetti Portela Alves, Marcelo Grossi Araújo, Flavia Vasques Bittencourt

**Affiliations:** 1 Universidade Federal de Minas Gerais (UFMG), Hospital das Clinicas, Departamento de Ortopedia e Traumatologia, Minas Gerais, MG, Brazil.; 2 Empresa Brasileira de Servicos Hospitalares (Ebserh), Belo Horizonte, Minas Gerais, Brazil.; 3 Universidade Federal de Minas Gerais (UFMG), Hospital das Clinicas, Departamento de Dermatologia, Minas Gerais, MG, Brazil.

**Keywords:** Gait Analysis, Foot, Reproducibility of Results, Análise da Marcha, Pé, Reprodutibilidade dos Testes

## Abstract

**Objective:**

To expand existing knowledge on gait parameters in a healthy population using a baropodometer and to ensure the reliability of the equipment.

**Methods:**

A cross-sectional study was conducted with fifty healthy adults aged 24 to 65 years. Parameters were collected from both sides over at least five valid steps. The Wilcoxon test was performed, and the Intraclass Correlation Coefficient (ICC) for static and dynamic measurements was obtained with 95% confidence and a p-value of 0.005.

**Results:**

The platform's reliability exceeded 0.7 for all parameters, both in static and dynamic analysis. All parameters showed a difference of less than 10% compared to the mean estimate across the five tests performed.

**Discussion:**

The platform's reliability showed moderate to high ICC values, indicating that, under similar hardware and software conditions, platforms from different manufacturers may produce comparable results. The ICC for PTI was lower than that of the other variables, possibly due to physiological differences in posture, which affect gait speed.

**Conclusion:**

The HS Tecnology manufacturer's baropodometer is a reliable tool for gait analysis in barefoot healthy individuals. ***Level of Evidence III; Cross-Sectional Study.***

## RESUMO

**Objetivo:**

Ampliar o conhecimento existente sobre os parâmetros da marcha em uma população saudável utilizando um baropodômetro e garantir a confiabilidade do equipamento.

**Métodos.:**

Estudo transversal com análise cinquenta adultos saudáveis, com idades entre 24 e 65 anos. Os parâmetros foram coletados de ambos os lados, em pelo menos cinco passos válidos. Teste de Wilcoxon foi realizado e o Coeficiente de Correlação Intraclasse (ICC) para as medições estáticas e dinâmicas foi obtido com 95% de confiança e valor de p de 0,005.

**Resultados.:**

A confiabilidade da plataforma foi superior a 0,7 em todos os parâmetros, tanto na análise estática quanto dinâmica. Todos os parâmetros apresentaram uma diferença inferior a 10% em relação à estimativa da média nos cinco testes realizados.

**Discussão.:**

A confiabilidade da plataforma mostrou valores de ICC moderados a altos, indicando que, sob condições de hardware e software similares, plataformas de diferentes fabricantes podem gerar resultados comparáveis. O ICC do PTI foi menor que o das outras variáveis, possivelmente devido a diferenças fisiológicas na postura, que afetam a velocidade da marcha.

**Conclusão.:**

O baropodometro de um fabricante Baroscan^®^ (HS Tecnology, Brasil) é uma ferramenta confiável para análise da marcha em indivíduos saudáveis descalços. ***Nível de Evidência III; Estudo Transversal.***

## INTRODUCTION

Gait analysis is part of many clinical and research protocols, and in order to perform it accurately and objectively, it is necessary to use equipment capable of performing kinematic or force measurements.^1–6^ During gait, body weight is transferred to the foot upon contact with the ground, starting at the heel and ending at the forefoot.^7–10^ Measuring ground contact pressure, contact time, and support area distribution provides a variety of information about body position, balance, and the interference of external loads.^4^ According to Rosário et al., the imbalance resulting from postural impairments can lead to functional overload, dysfunction, degeneration, or even clinical problems related to incapacitating pain.^4^ The presence of high pressure peaks in certain regions of the foot has been widely studied as an important causal factor for various diseases and deformities, especially those located in the functional complex of the foot and ankle.^11–13^

The baropodometer is an advanced force platform used to measure plantar pressure in both static and dynamic positions during gait analysis.^14^ To do this, dedicated hardware is required for proper data collection and compatible software to process this data and produce images similar to those of a podoscope.^14–20^ This technique provides direct and indirect information about the patient's position when standing still, walking, or even running.^18^ Baropodometric analysis is capable of assessing foot dysfunction and can map and record plantar pressure peaks and changes in body balance, enabling the analysis of anomalies.^20^ During static assessment, pressure values, area, and center of pressure (COP) deviations can be obtained.^9–11^ Dynamic gait analysis allows the collection of data on load distribution during walking, measurement of peak pressure, contact area, and contact time with each region of the foot in relation to the ground.^9–11^ Thus, this tool is useful for detecting areas at risk of pain, calluses, or even foot deformities, as well as biomechanical anomalies of the foot, pelvis, and spine.^3,4,13^ On the other hand, it is also frequently used in the manufacture of custom orthopedic insoles or footwear to accommodate deformities or modify pressure points for the treatment of pain, especially when located in the foot or ankle.^13^

The peak pressure variable (PP) is calculated using the vertical component of the ground reaction force (GRF). To this end, pressure sensors essentially act as force transducers, measuring the force acting on a surface of known area.^3,5-8,14^

There are advantages to using a baropodometer in clinical practice or for research purposes, as it enables non-invasive analysis of gait, posture, and body balance with high accuracy. However, the accuracy and repeatability of absolute values obtained through baropodometric assessment have been questioned in previous studies examining different pressure systems. These studies compared various devices and performed some hardware technology comparisons to confirm the reproducibility and reliability of the platforms, indicating that comparative studies are needed due to the variability of results obtained with different technologies.^2–4^

The platform technology depends largely on the manufacturer, which can result in a variety of hardware resolutions, sensor types, sampling rates, and detectable pressure ranges. These possible variations between manufacturers can impact the results of force or time measurements that plantar pressure exerts on the device, especially in research contexts. Researchers and clinicians may need to compare data collected from different platforms due to equipment cost, availability in each location, changes in technology over time, or even when used together in multicenter studies to combine data sets from separate studies or clinics.^3,5-8,14^

There are several companies worldwide that manufacture plantar pressure platforms, but only a few have had their equipment tested for accuracy and reliability. Some studies comparing hardware and software with similar specifications from different manufacturers have shown moderate to high correlations, with Intraclass Correlation Coefficients (ICC) greater than 0.7 in comparisons between evaluators.^4,5,7,8,15,18^ Therefore, it is crucial to verify the reproducibility of similar hardware and software technologies, even when they come from different manufacturers around the world, to ensure that they provide consistent and accurate results. Ensuring reproducibility can significantly enhance the utility of baropodometers, particularly in developing regions where access to previously validated or high-quality equipment may be limited. By confirming the accuracy and consistency of these tools, researchers and clinicians in such fields can use them with greater confidence in their studies and practices.

Currently, some plantar pressure measurement systems on the market have validated their results as reliable and repeatable, such as platform-based systems (e.g., Novel Emed^®^, TekScan MatScan^®^, Medicapteurs S-Plate^®^, RS-Scan Footscan^®^, Poprint^®^ and Podoprint^®^) (1,3,18,19) and systems based on insoles or footwear (e.g., Novel Pedar^®^, TekScan F-Scan^®^, RS-Scan Insole^®^, WalkinSense^®^, and IBV Biofoot^®^).^5–8,13^ These plantar pressure measurement devices have also proven to be reliable tools for quantifying dynamic plantar pressures. However, most of these systems are manufactured in the United States and Europe, resulting in high acquisition and maintenance costs, especially in developing countries. Additionally, each system typically requires specific software for data processing, which may not be readily available in all regions of the world. Therefore, it is essential to evaluate the validity, reliability, and effectiveness of locally available pressure systems to ensure their suitability for clinical use and large-scale research.

The objective of this study is to evaluate the accuracy and repeatability of the national platform and expand existing knowledge about gait parameters in a healthy population.

## MATERIALS AND METHODS

### Participants

The study was approved by the Research Ethics Committee (CEP) of the Federal University of Minas Gerais (UFMG CAAE 27968619.9.0000.5149. After CEP approval, 50 healthy adults (25 men and 25 women) were invited to participate in the study. Participants were aged between 24 and 65, with body mass indices (BMIs) ranging from 18 to 37. All participants were physically active, had no comorbidities, pain, or limitations that could affect their walking ability, and signed the informed consent form.

### Platform

The Baroscan^®^ (HS Tecnology, Brazil) used in this study measures 680 x 510 x 10 mm and weighs 2.2 kg. It was equipped with 4096 resistive sensors, each measuring 10 x 10 mm, covering a total contact area of 500 x 500 mm. The platform is capable of measuring static and dynamic pressure between 0.4 and 100 N/cm^2^, with a maximum sampling frequency of 200 Hz. The manufacturer calibrated the equipment within one year before data collection.

### Warm-up Period and Data Collection

Participants were instructed to walk barefoot, at their own pace and speed, for five minutes on a five-meter-long walkway, with the baropodometric plate positioned in the first third of the walkway. After this warm-up period, data were collected from the left and right feet, with a minimum of five steps per foot. Only tests in which the foot made contact with the entire platform were considered valid. Five valid tests were recorded for each participant in the initial session and again after a two-week interval. All data were subjected to statistical analysis.

### Statistical Analysis

Descriptive statistics were calculated for each plantar pressure parameter, including static and dynamic tests across the entire surface of the foot. The data were analyzed using SPSS statistical software (IBM®, Chicago IL). To assess reproducibility, the Wilcoxon test was performed, and the ICCs for both static and dynamic measurements were calculated with a 95% confidence interval and a significance level of p < 0.005.

## RESULTS

The study participants consisted of 25 healthy men and 25 healthy women who agreed to participate. The average age was 48.9 years (ranging from 24 to 65 years). The average body mass index (BMI) of the subjects was 25.32 (ranging from 18.31 to 37.55).

During static analysis, the pressure values found in each variable were summarized (Table 1), and ICC values greater than 0.7 were obtained for all parameters, such as PP (0.705) and MP (0.748), with a 95% confidence interval and p<0.005 (Table 2).

**Table 1:** Baropodometric gait parameters in healthy subjects during static analysis at the initial appointment (E1) and the second appointment two weeks later (E2) are presented as medians and quartiles. (n = 50).

Parameters	E1	E2
Peak of pressure kPa (D)	1.29 (1.06 - 1.56)	1.39 (1.15 - 1.66)
Peak of pressure kPa (E)	1.27 (1.01 - 1.64)	1.41 (1.06 - 1.66)
Mean pressure kPa (D)	0.36 (0.30 - 0.40)	0.39 (0.31 - 0.43)
Mean pressure kPa (E)	0.35 (0.28 - 0.41)	0.38 (0.32 - 0.44)
Contact area (cm2)	83.62 (67.14 - 94.15)	79.66 (68.36 - 93.99)

**Table 2:** Static analysis comparing ICC, IC, and p-value of mean parameters for intra-platform correlation at the first appointment with the second appointment after two weeks apart. (n = 50).

Static parameters	ICC	IC 95%	p-value
Peak of pressure (kPa)	0.705	(0.533; 0.821)	0.000
Mean pressure (kPa)	0.748	(0.595; 0.849)	0.000
Contact area (cm2)	0.801	(0.673; 0.882)	0.000

ICC- Intraclass correlation coefficient. CI – Confidence interval. *p-value* – statistical significance when p<0.05.

The dynamic analysis is summarized in Tables 3 and 4, which present the pressure values found in this population for each test performed. The results also showed ICC values greater than 0.7 for all parameters: peak dynamic pressure (DPP) (0.828), mean dynamic pressure (DMP) (0.82), pressure time index (PTI) (0.797), and contact time (CT) (0.761) with a 95% confidence interval and p<0.005. (Table 5) The reliability of the platform exceeded 0.7 in all parameters analyzed, both in static analysis (Table 2) and dynamic analysis (Table 5). All parameters showed a difference of less than 10% from the unbiased estimate of the mean within the five tests performed.

**Table 3:** Baropodometric gait parameters in healthy subjects during dynamic analysis at the initial appointment (trials 1 to 5) are presented as median and quartiles (n = 50).

Parameters	1	2	3	4	5
Peak of pressure (kPa/cm^2^)	3.34 (2.8 - 4.31)	3.48 (2.92 - 4.35)	3.49 (2.95 - 4.68)	3.54 (3.13 - 4.50)	3.5 (3.06 - 4.49)
Mean pressure (kPa/cm^2^)	0.96 (0.81 - 1.05)	0.88 (0.8 - 0.97)	0.87 (0.79 - 0.99)	0.93 (0.79 - 0.98)	0.91 (0.79 - 0.98)
Integral pressure time (kPa s/cm^2^)	0.27 (0.22 - 0.35)	0.27 (0.21 - 0.37)	0.27 (0.24 - 0.35)	0.29 (0.23 - 0.35)	0.27 (0.24 - 0.34)
Time of contact (ms)	597.5 (491.25 - 682.5)	530 (447.5 - 625)	507.5 (430 - 591.25)	480 (398.75 - 547.5)	472.5 (383.75 - 540)

Trials 1, 2, 3, 4, 5: median and quartiles of each plantar pressure analysis during initial appointment.

**Table 4:** Baropodometric gait parameters in healthy subjects during dynamic analysis at the second appointment two weeks later (trials 1 to 5) are presented as median and quartiles (n = 50).

Parameters	1	2	3	4	5
Peak of pressure (kPa/cm^2^)	3.38 (2.85 - 3.95)	3.31 (2.85 - 4.10)	3.48 (3.02 - 4.03)	3.45 (3.02 - 4.56)	3.45 (2.92 - 4.37)
Mean pressure (kPa/cm^2^)	0.87 (0.80 - 0.97)	0.93 (0.80 - 1.03)	0.91 (0.84 - 1.10)	0.96 (0.87 - 1.06	0.93 (0.86 - 1.06)
Integral pressure time (kPa s/cm^2^)	0.27 (0.22 - 0.31)	0.26 (0.22 - 0.33)	0.27 (0.22 - 0.34)	0.29 (0.22 - 0.36)	0.29 (0.22 - 0.39)
Time of contact (ms)	550 (525 - 650)	570 (475 - 661.25)	532.5 (423.75 - 627.5)	497.5 (395 - 558.75)	490 (380 - 546.25)

Trials 1, 2, 3,4,5: median and quartiles of each plantar pressure analysis at second appointment.

**Table 5:** Dynamic analysis comparing ICC, IC, and p-value of mean parameters for intra-platform correlation at the first appointment with the second appointment after two weeks apart. (n = 50).

Dynamic parameters	ICC
Peak of pressure (kPa)	0.828
Mean pressure (kPa)	0.82
Integral pressure time (kPa s/cm^2^)	0.797
Time of contact (ms)	0.834

ICC – intraclass correlation coefficient. CI – Confidence interval.

* *p-value* – statistical significance when p<0.05.

The pressure values obtained in both assessments, including static and dynamic gait assessments on the two assessment days, are summarized in Tables 1, 3, and 4 for this population of healthy individuals.

## DISCUSSION

Foot pressure measurements are part of many clinical and research protocols, and several companies currently manufacture and distribute foot pressure platforms worldwide. Platform technology is specific to each manufacturer, and suppliers can offer a wide variety of hardware resolutions, different types of sensors, sampling rates, and pressure detection ranges. For many researchers, especially in some developing countries, previously validated baropodometry devices may not be available for purchase, or their maintenance costs may be prohibitive. As technology continues to advance, even in developing countries, a growing number of new manufacturers are producing baropodometry equipment with specifications comparable to those of established, high-quality, internationally validated models. The availability of this new type of domestically produced equipment could expand its daily use in medical offices and broaden its application in clinical research. To this end, even under similar hardware and software specifications, researchers must ensure the validity and comparability of results between available equipment to enable meaningful comparisons in the literature.

Furthermore, this study was conducted in a healthy population, which increases the amount of information on gait analysis data and helps to understand the variability of non-pathological thresholds for static and dynamic gait. Therefore, the objective of this study was to evaluate the accuracy and repeatability of a nationally manufactured baropodometry platform (XXX), with hardware and software specifications similar to those of internationally validated models, using a healthy population. This assessment is crucial for understanding the variation in different technologies used in clinical and research settings, as well as for increasing knowledge about possible non-pathological variations in gait analysis.

The results demonstrate that the variables PP, MP, and TC presented ICC values greater than 0.8. These results are consistent with previous reports, which have shown high reliability for these variables across different brands and manufacturers.

In this study, the PTI variable values presented an ICC value of 0.797. Although this is the lowest ICC among the variables measured, it still indicates moderate reliability. This result is lower than that of PP, TC, and MP, which contrasts with previous studies that found higher ICCs for PTI. According to Murphy et al.^12^, differences in pressure measurements can be attributed to small, unavoidable physiological changes that may occur during non-pathological walking, such as changes in speed, body position, and muscle activity, which can affect load distribution and ultimately interfere with some measured parameters. PTI describes the cumulative effect of pressure over time on a specific area of the foot, providing the total load exposure of an area of the sole with each step during walking. The moderate reliability of the PTI can be attributed to these physiological changes, as this variable is closely related to walking speed. Previous studies suggest that reducing the average number of attempts can decrease gait variability and enhance the accuracy of results. However, even with five attempts, some physiological changes during walking may be present, ranging from fluctuations in speed to minimal postural adjustments, which could eventually interfere with the results, as demonstrated in a study by Hafer et al.^7^ Many studies have demonstrated comparable reliability within a session and between sessions of different pressure platforms or insoles, both in healthy subjects and in groups with specific pathologies, with ICC values greater than 0.7. Similar results were found in our study. Overall, the reliability results of the platform demonstrate moderate to high ICC values, indicating that plantar pressure measurements using this equipment are consistent. This suggests that, under similar *hardware* and *software* specifications, different platforms from various manufacturers could produce comparable results.

Additionally, it is crucial to collect data on healthy populations to facilitate future comparisons with populations that may have gait and balance disorders. Previous studies, such as that by Cordeiro et al.^19^, have already demonstrated that even with pressures lower than 195 kPa, some pathologies can lead to the development of ulcers or deformities in the feet, thus highlighting the need to relate healthy populations to the various gait parameters obtained with this type of equipment.

## CONCLUSION

Generally, baropodometers produced by domestic manufacturers are reliable tools, with ICC values above 0.7 in the parameters analyzed to assess plantar pressure distribution in both static and dynamic measurements in a healthy population.
